# RAG: An update to the RNA-As-Graphs resource

**DOI:** 10.1186/1471-2105-12-219

**Published:** 2011-05-31

**Authors:** Joseph A Izzo, Namhee Kim, Shereef Elmetwaly, Tamar Schlick

**Affiliations:** 1 Department of Chemistry, New York University, 100 Washington Square East, New York, NY 10003, USA; 2 Courant Institute of Mathematical Sciences, New York University, 251 Mercer Street, New York, NY, 10012, USA

## Abstract

**Background:**

In 2004, we presented a web resource for stimulating the search for novel RNAs, RNA-As-Graphs (RAG), which classified, catalogued, and predicted RNA secondary structure motifs using clustering and build-up approaches. With the increased availability of secondary structures in recent years, we update the RAG resource and provide various improvements for analyzing RNA structures.

**Description:**

Our RAG update includes a new supervised clustering algorithm that can suggest RNA motifs that may be "RNA-like". We use this utility to describe RNA motifs as three classes: existing, RNA-like, and non-RNA-like. This produces 126 tree and 16,658 dual graphs as candidate RNA-like topologies using the supervised clustering algorithm with existing RNAs serving as the training data. A comparison of this clustering approach to an earlier method shows considerable improvements. Additional RAG features include greatly expanded search capabilities, an interface to better utilize the benefits of relational database, and improvements to several of the utilities such as directed/labeled graphs and a subgraph search program.

**Conclusions:**

The RAG updates presented here augment the database's intended function - stimulating the search for novel RNA functionality - by classifying available motifs, suggesting new motifs for design, and allowing for more specific searches for specific topologies. The updated RAG web resource offers users a graph-based tool for exploring available RNA motifs and suggesting new RNAs for design.

## Background

The RAG (RNA-As-Graphs) web resource was launched in 2004 to classify and catalogue all possible RNA 2D topologies, including existing and hypothetical motifs http://www.biomath.nyu.edu/rna[1,2]. RAG's construction was motivated by the increasing importance of RNAs, structurally diverse molecules with significant regulatory roles including protein synthesis, transcriptional regulation and other integral biological functions [3-8]. Many databases have been designed for classifying existing RNAs. These include the NDB (nucleic acid database) [9,10], Rfam (RNA families with consensus secondary structures) [11,12], SCOR (structural classifications of RNAs) [13,14], RNA Strand (secondary structures) [15], and Pseudobase++ (RNAs containing pseudoknots) databases [16,17].

To aid these classification efforts, RAG was designed to enumerate and classify all possible RNA topologies including both existing and missing motifs (Tables 1 and 2) [1,2]. Specifically, we utilized a graphical representation of RNA secondary structure using elements of graph theory, including tree and dual graphs, adjacency matrices, and Laplacian eigenvalues. By representing secondary structures as tree and dual graphs and classifying them by their vertex number and eigenvalue spectrum, we hoped to facilitate the search for novel RNA motifs. RNA graphs were enumerated by both analytical and exhaustive computational approaches, and classified into either "existing" graphs - those found in RNA databases of solved structures or comparative structure analysis - and "missing" graphs - motifs that had not yet been found. RAG has been used for classification and prediction of non-coding RNAs [18-21], modification of RNA graph representations into labeled and directed graphs [18,22-24], and RNA structural analysis [25-29]; see recent reviews [30,31] and the Discussion section on RAG's application to RNA design.

**Table 1:** The current number of RNA tree topologies, divided into existing and yet unreported, the latter subdivided into RNA-like and non-RNA-like by PAM and a supervised clustering algorithm (*k*-NN) based on existing RNA

V, vertex no.	Existing	RNA-like		Non-RNA-like		Total
			
		PAM	*k*-NN	PAM	*k*-NN	
2	1	0	0	0	0	1

3	1	0	0	0	0	1

4	2	0	0	0	0	2

5	3	0	0	0	0	3

6	6	0	0	0	0	6

7	9	2	2	0	0	11

8	15	3	5	5	3	23

9	11	22	32	14	4	47

10	10	60	87	36	9	106

Total	**58**	**87**	**126**	**55**	**16**	**200**

**Table 2:** The current number of RNA dual topologies, divided into existing and yet unreported, the latter subdivided into RNA-like and non-RNA-like by PAM and a supervised clustering algorithm (*k*-NN) based on existing RNA

V, vertex no.	Existing	RNA-like		Non-RNA-like		Total
			
		PAM	*k*-NN	PAM	*k*-NN	
2	3	0	0	0	0	3

3	8	2	0	2	0	8

4	17	8	3	11	10	30

5	18	63	45	36	45	108

6	12	307	239	185	243	494

7	6	1,604	1,139	783	1,243	2,388

8	3	8,777	5,275	3,407	6,906	12,184

9	4	25,810	9,957	12,785	28,634	38,595

Total	**71**	**36,571**	**16,658**	**17,209**	**37,081**	**53,810**

In 2004, we expanded this work on RNA graphs to predict sequences that fold into 10 candidate topologies based on a modular approach using functional submotifs taken from existing structures [32]. That work included a clustering analysis that partitioned all non-existing RNA topologies with 3 and 4 vertices into two classes: "RNA-like" and "non-RNA-like." A recent search of the experimentally verified non-coding RNA databases such as the Rfam database indicated that 5 of the 10 designed candidate topologies now exist in nature. Moreover, they are found in multiple RNA families (see the Discussion section on statistics of current existing topologies).

Since our 2004 work, RNA databases have grown significantly. For example, the RNA family database (Rfam), which displays consensus secondary structures for different families of RNA, had 367 families in 2004, and now contains 1,372 families (database 9.1, December 2008) [11,12]. The RNA Strand database, which catalogues existing secondary structures from various structure databases including NDB, PDB, and others, now holds 4,666 structures that have been determined from a variety of theoretical and experimental methods such as comparative sequence analysis, NMR data, and X-Ray crystallography [15].

This vast increase in the amount of RNA structural data provides an opportunity to update RAG and to compare our earlier "RNA-like" and "non-RNA-like" classifications to newly discovered RNA. Further, we propose an improved classification of RNA-like and non-RNA-like topologies using a supervised clustering algorithm based on existing RNAs. In addition, we implement various improvements to our RAG web resource such as expanded search tools and a user-friendly interface.

## Construction and Content

The original RAG database was designed with the following elements: graphical representations of RNA secondary topologies; Laplacian eigenvalues for quantitative description of RNA graphs; prediction of candidate RNA topologies using a clustering algorithm; and a program for converting secondary structures into RNA tree graphs. In the updated version, we improve upon the database's functionality, apply a supervised clustering algorithm to suggest candidate topologies, and compile the new RNA structures into a user-friendly interface.

### RNA graphical representation

**Figure 1:**
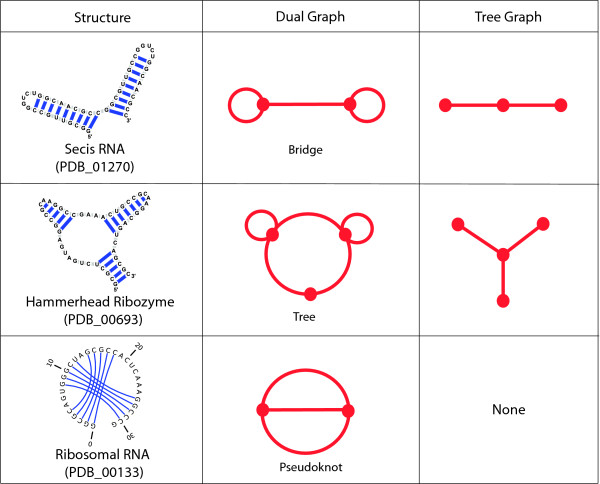
**A depiction of how to represent RNA secondary structures as both dual and tree graphs**. All non-pseudoknot structures (the first and second rows) can be translated into both dual and tree graphs while a pseudoknot structure (the third row) can only be depicted by a dual graph. Each RNA in the first column exists in nature as a whole (Hammerhead ribozyme) or as a part of domain (mRNA - Secis element and rRNA); their RNA Strand IDs are in parenthesis.

We utilize both tree and dual graphs to represent RNA structure. Tree graphs offer a general description of RNA structures, while dual graphs are more specific and allow us to represent pseudoknot structures as well (see Figure 1). The rules for expressing 2D RNA structure as tree graphs are as follows [33-35]:

1) The 3' and 5' ends of a helical stem constitute a single vertex (•).

2) An RNA stem with more than one complementary base pair is considered an edge (-); complementary base pairs are considered to be AU, GC, and the special case of the GU wobble.

3) A bulge, hairpin, or internal loop is a vertex (•) if there is more than one unpaired nucleotide or non-complementary base pair.

4) An RNA junction is considered to be a vertex (•).

The rules for transforming RNA 2D topologies into dual graphs are the opposite of those for tree graphs, namely:

1) The 3' and 5' ends do not have any representation.

2) An RNA stem with more than one complementary base pair is represented as a vertex (•).

3) An edge (-) represents any single strand that has more than one unpaired nucleotide and occurs in segments connecting secondary elements of the 2D structure such as bulges, internal loops, junctions, and stems.

The main advantage of such coarse-grained representations is their reduced space compared to the atomic-level structural space or RNA sequence space. This facilitates addressing many problems in RNA structure and allows cataloging of a finite set of graphs to represent all existing and hypothetical RNA structures.

### Topological descriptors of RNA graphs: Laplacian eigenvalues

The graph connectivity is described by a Laplacian matrix constructed from the adjacency and degree matrices of each graph; the full eigenvalue spectrum of the Laplacian matrix can then be computed [1,32]. The spectrum is useful for differentiating between RNA graphs; the number of zero eigenvalues indicates the number of disconnected elements of the graph and the value of the second smallest eigenvalue (λ_2_) is a measure of the complexity of the graph (a linear RNA molecule has a smaller λ_2 _value than a branched molecule). Thus, two graphs with differing spectrum are dissimilar, though the converse is not true [32]. Since motif complexity is indicated by the second smallest eigenvalue (λ_2_) of the Laplacian matrix, the ordering of λ_2 _values for topologies within each vertex number (V) is used in RAG to derive a motif identification number (ID) to distinguish the topological complexities among topologies of a certain vertex number. In RAG, all possible tree graphs up to 10 vertices and dual graphs up to 9 vertices have been enumerated and classified through this system of (V, ID, λ_2_).

### Predicted topologies: RNA-like and non-RNA-like graphs

We employed a clustering analysis in our previous work [1,32] to determine the potential for our enumerated topologies to be discovered in nature, i.e., to be "RNA-like". The Partitioning Around Medoids (PAM) [36] was used in conjunction with a linear transformation of the Laplacian eigenvalue spectrum of each topology to separate RNA graphs into two distinct classes as follows:

1) The values from the Laplacian eigenvalue spectrum are transformed through a linear regression into two variables - the intercept (α) and the slope (β). Specifically, we transform the non-zero Laplacian eigenvalues for a V-vertex graph (λ_2_, λ_3_,..., λ_V_) into two variables (α, β) by applying the least-squares method regarding the index (2, 3,...,V). The slope β and the intercept α represent the average spacing between eigenvalues and the second eigenvalue (λ_2_) adjusted by β, respectively.

2) (α, β) is normalized to (α, V*β), because β decreases with the vertex number (V), and thus, V*β can be considered graph-size independent.

3) A distance matrix is created from the variables (α, V*β) corresponding to the pair-wise distance between all RNA graphs.

4) Using the distance matrix in Step 3, PAM is applied to cluster all RNA graphs into two groups.

Briefly, the PAM clustering algorithm for two clusters functions by selecting in turn two representatives (also called medoids) and assigning each member into the closest group among two groups based on the distance matrix. These steps are repeated until the resulting two groups of data points have both maximum dissimilarity between groups and maximum similarity within groups. This method allowed us to classify hypothetical topologies as "RNA-like" or "non-RNA-like"; the former group of topologies is considered more likely to be found in nature.

Since PAM does not treat existing data with greater weight, we apply here an improved statistical method, the *k*-nearest neighbor (*k*-NN) algorithm [37,38], to classify missing motifs as RNA-like or non-RNA-like based on training data of known RNA topologies. Briefly, *k*-NN is an instance-based supervised classification algorithm for classifying objects based on *k *closest training data in the description space: an object is classified by a majority vote of its neighbors, with the object being assigned to the class most common among its *k *nearest neighbors. Here, neighbors are defined by the Euclidian distance between graph descriptors - the transformation of Laplacian eigenvalues. Typically, the parameter *k *is a small number from 1 to 5. For our training sets, we use partial sets of existing and missing graphs with 3-4 vertices for dual graphs and 3-8 vertices for tree graphs. We found that *k *= 3 yields reasonable accuracy when the error rate is calculated by cross-validation analysis for *k *= 1 to 5 (see Table 3). The assessment of *k*-NN versus PAM is elaborated separately in the following Discussion section.

**Table 3:** Cross-validation (CV) results for dual graphs with *k*-NN (*k *= 1 to 5) and PAM

Training Set (2010)		Error Rate
	
Dual graphs (V)	Method	(10-fold CV) [%]
3-4	1-NN	8
	
(25 existing and 25 missing graphs)	2-NN	6
	
	3-NN	8
	
	4-NN	6
	
	5-NN	10
	
	PAM	34

3-5	1-NN	14
	
(43 existing and 43 missing graphs)	2-NN	12
	
	3-NN	15
	
	4-NN	13
	
	5-NN	14
	
	PAM	26

3-6	1-NN	13
	
(55 existing and 55 missing graphs)	2-NN	13
	
	3-NN	13
	
	4-NN	13
	
	5-NN	13
	
	PAM	26

3-7	1-NN	13
	
(61 existing and 61 missing graphs)	2-NN	13
	
	3-NN	13
	
	4-NN	13
	
	5-NN	12
	
	PAM	31

3-8	1-NN	13
	
(64 existing and 64 missing graphs)	2-NN	14
	
	3-NN	12
	
	4-NN	13
	
	5-NN	13
	
	PAM	33

3-9	1-NN	13
	
(68 existing and 68 missing graphs)	2-NN	13
	
	3-NN	13
	
	4-NN	13
	
	5-NN	12
	
	PAM	36

### RNA Matrix: a computer program to convert RNA 2D topology to a tree graph

RAG contains the RNA Matrix program to assist structural and functional identification of RNA motifs. It converts a user-supplied secondary structure file (in 'ct' format) into its graphical representation. Essentially, our RNA Matrix program converts a tree secondary structure into an adjacency matrix through the following two steps: (1) the secondary structure file ('ct' file format) is used to define paired (P) and unpaired (U) regions of the RNA sequence. Each U region is associated with a vertex label (1, 2, 3, ...). Regions with the same label belong to the same structural elements (e.g., junctions, bulges). Identifying the U and P regions involves applying the RNA graph rules and also requires careful consideration of possible secondary structure configurations (e.g., where the chain ends occur); (2) the adjacency graph vertices are assigned by following the connecting arrows (from left to right). For dual graphs, the roles of U and P regions are reversed. RNA Matrix calculates the RNA graph's topological characteristics (vertex number, eigenvalues, order of junctions or degree of vertices, etc). Such information directs the user to the corresponding existing (or hypothetical) RNA motif in the database, with links to other RNA sequence, structure (2D and 3D) and function databases. Our RAG update introduces three significant improvements to the RNA Matrix program: first, we have now automated the classification of dual graph as well as tree graph topologies; second, we have extended the limit of 200 nt to 1000 nt and of 10 vertices to any number for tree graphs; third, we added a function to specify the direction for the graphs (5' to 3') and list common subgraphs between two RNA secondary structures. To label vertices, RNA Matrix compares the adjacency matrix of given structures to a set of standard adjacency matrices corresponding to our labeled graphs (visible on the RAG website). Subgraphs are determined by permuting all square submatrices of the structures' adjacency matrices to identify smaller graphs shared by each structure.

## Utilities and Discussion

### RNA database searches

We determine and classify tree and dual graphs of available secondary structures according to our topological descriptors (Tables 1 and 2). To accomplish this, we use secondary structure information from three comprehensive RNA databases to produce RNA graphs: the Rfam database, which contains the sequence alignments and consensus secondary structures of RNA families; the Pseudobase++ database, a catalogue of pseudoknot structures; and the RNA Strand database, which collects RNA secondary structures from many databases such as the RCSB Protein Databank, Nucleic Acid Database, and others. We use the following criteria for converting database structures to RNA graphs: tree graphs and dual graphs are limited to 10 vertices and 9 vertices, respectively; only RNAs of 200 nt or less are considered. Additionally, only pseudoknot-containing structures are considered by the dual graph representation, because tree graphs cannot represent pseudoknots. The non-pseudoknot structures are represented by both tree and dual graphs.

We consider natural RNAs whose structures are solved by experimental methods, such as NMR or X-ray crystallography or identified by comparative analysis to be existing RNAs. All comparative structures are currently derived from the Pseudobase++, Rfam and RNAStrand databases, while solved structures are from NDB, PDB, and Pseudobase++. The secondary structure prediction by homology modeling exploits multiple RNA sequences to infer accurate conserved secondary structures: the homologous sequences are aligned to determine conserved residues and the common secondary structure is detected by the co-variation of base pairs [11]. In our earlier RAG, we also took account into structures from comparative structure analysis. Indeed, many comparatively analyzed structures have been verified by mutagenesis or chemical probing (see Table S1 in Additional file 1 for the list of Rfam IDs of families whose comparatively analyzed secondary structures are confirmed by mutagenesis or structure probing). Synthetic RNAs are not regarded as existing topologies because they were not found in nature, but designed to have a specific structure or function *in vitro*. Still, in the RAG resource, we show a list of synthetic RNA structures determined by experimental methods (X-ray crystal structures or NMR), as listed in NDB, PDB or Pseudobase++.

We use the RNA Matrix program (available on our website for single-molecule use) to rapidly deduce tree graph topologies from the available .ct files of RNA Strand and dual graph topologies from the .bpseq secondary structure files of Pseudobase++. We manually determine the topologies for the RNA families of Rfam, if each topology can be represented by a tree graph of 10 vertices or less or a dual graph of 4 vertices or less, because the database does not currently offer secondary structure files. The tree and dual graph topologies are then classified by motif class (existing, RNA-like, or non-RNA-like) and the method used to determine the structure.

### Statistics of current existing topologies

In our 2004 work, 200 tree graph topologies were enumerated for motifs up to 10 vertices, and 53,810 dual graph topologies were enumerated for motifs up to 9 vertices (see the last columns of Tables 1 and 2). RNAs corresponding to 24 of the tree graph topologies and 29 of the dual graph topologies were found in structural databases of the time. These topologies were placed into an "existing" classification. The remaining topologies were considered "missing," and subdivided into RNA-like or "non-RNA-like" classifications by predictions made using PAM clustering. Because three of the graphs reported as existing in the 2004 RAG database could not be located currently (they were predicted by Mfold but not confirmed experimentally), the 2004 existing data as reported has been corrected: the set of 24 existing trees have now been reduced to 21 tree graphs. See Table S2 in Additional file 1 for these corrections.

**Figure 2:**
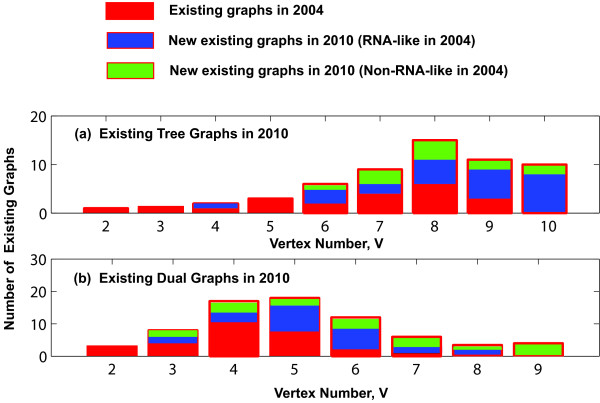
**The current number of existing (a) tree graphs and (b) dual graphs in each graph vertex number (from 2 to 10 for tree graphs and from 2 to 9 for dual graphs) in the updated RAG database**. Each bar is divided by the topological classifications that were constructed in 2004 (existing, RNA-like, and non-RNA-like) which are represented as red, blue and green, respectively. Since the launch of RAG in 2004, more than twice as many topologies have been identified, most of which have been confirmed from RNA-like topologies (see the second column of Tables 1 and 2 for the distribution of existing motifs in each vertex number from 2 to 10 for tree graphs and from 2 to 9 for dual graphs, respectively).

Our current database searches for solved and comparatively-analyzed structures have identified more than twice as many topologies than were found in our 2004 searches: 58 tree graphs (21 in 2004) and 71 dual graphs (29 in 2004). The first column of Tables 1 and 2 shows the current numbers of existing tree and dual topologies with vertex numbers 2 to 10 for tree graphs and 2 to 9 for dual graphs, respectively. There are 37 new tree and 42 new dual existing topologies that were previously classified as missing topologies. Figure 2 shows the breakdown of current existing tree and dual topologies for each vertex number by their status in 2004 (existing, RNA-like, or non-RNA-like). For tree graphs, 25 newly-confirmed topologies (blue in Figure 2) were classified as RNA-like in 2004 and 12 were classified as non-RNA-like in 2004 (green in Figure 2). For dual graphs, 24 newly-confirmed topologies were classified as RNA-like in 2004, and 18 were classified as non-RNA-like topologies in 2004. Because these results clearly leave room for improvement, we apply here a new clustering approach, supervised clustering, which depends more strongly on existing RNA (see also the previous subsection on predicted topologies).

**Figure 3:**
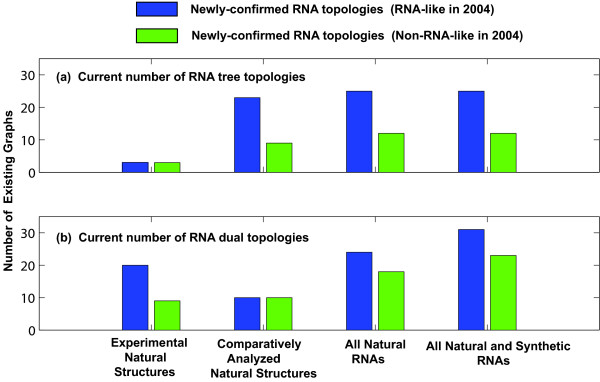
**The current number of tree graphs (a) and dual graphs (b) which are newly confirmed according to method of discovery - experimental (solved) natural structures, comparatively analyzed natural structures, all natural structures, and all RNAs (including both national and synthetic structures) in the updated RAG database**. Each number is divided by the topological classifications in 2004 (RNA-like and non-RNA-like) which are represented as blue and green, respectively.

For both tree and dual graphs, the accuracy of our predictions of RNA-like motifs depends on the method of structure discovery. Figure 3 shows the findings from newly-confirmed topologies belonging to structures determined by different methods: experimentally solved natural RNAs, comparatively-analyzed natural RNA sequences, and synthetically designed RNAs. Results from each method are shown by their classification in 2004: RNA-like (blue in Figure 3) and non-RNA-like (green in Figure 3). For tree graphs, when comparing RNA-like vs. non-RNA-like classifications in 2004, the numbers of topologies are 3 vs. 3 for solved structures, 23 vs. 9 for comparatively analyzed structures, and 2 vs. 1 for synthetic structures. For dual graphs, the numbers are 20 vs. 9 for solved structures, 10 vs. 10 for comparatively analyzed structures, and 14 vs. 6 for synthetic structures.

In particular, for the experimental structures, the dual graph has a much higher prediction rate (20 vs. 9, the first bar plot in Figure 3, lower) compared to tree graphs (3 vs. 3, the first bar plot in Figure 3, upper). This is because of the degeneracy of tree representations (i.e., different secondary structures can be represented as same tree topologies) and the higher sample size of solved dual graph structures (527) in contrast to solved tree structures (392) due to the addition of pseudoknot structures from Pseudobase++. However, as shown in the last two bar plot in Figure 3 (for all natural RNAs and all RNAs), though PAM clustering predictions are reasonable, there remains much room for improvement, as we discuss below. Note that there is some overlap among these topologies between different methods of discovery, and therefore the numbers are not additive for all natural RNAs (both experimental and comparative structures) and all RNAs (both natural and synthetic RNAs) as shown in the last two columns of Figure 3.

**Figure 4:**
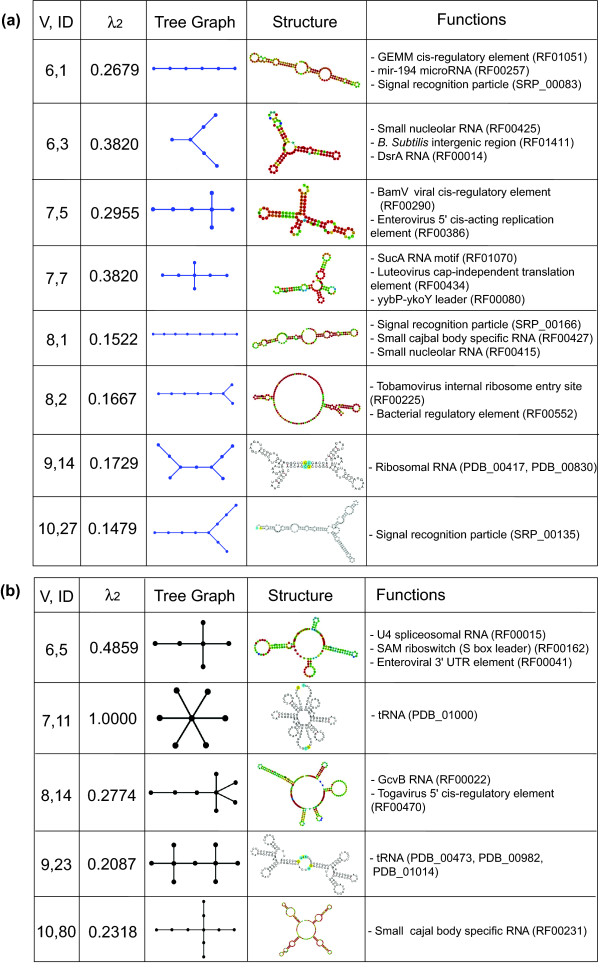
**Examples of newly confirmed RNA tree graphs from RNA-like (a) or non-RNA-like (b) graphs classified in the 2004 RAG database**. The vertex number/ID (first column) and the second smallest eigenvalue (second column) are shown for each RNA tree graph (third column). RNA secondary structures and their functions are shown in the fourth and fifth columns.

**Figure 5:**
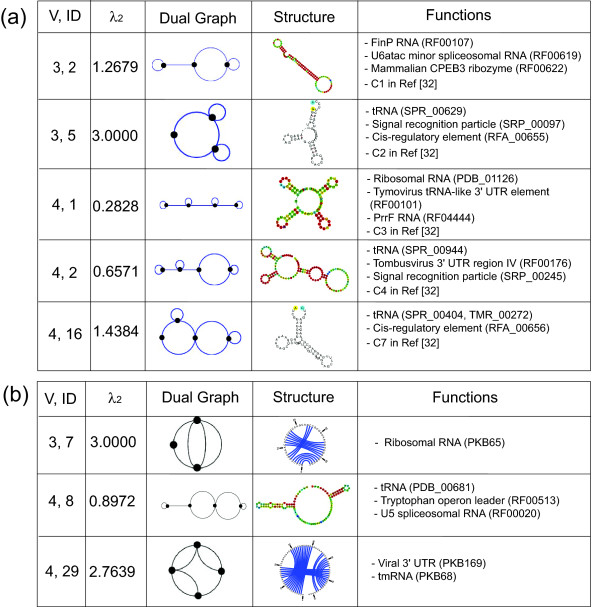
**Examples of newly confirmed RNA dual graphs from RNA-like (a) or non-RNA-like (b) graphs classified in the 2004 RAG database**. The vertex number, ID (first column) and the second smallest eigenvalue (second column) are shown for each RNA tree graph (third column). RNA secondary structures and their functions are shown in the fourth and fifth columns. In (a), the five identified RNAs that correspond to our candidate topologies with 3 and 4 vertices in 2004 using PAM [32] are shown (C1, C2, C3, C4 and C7).

RNAs which were classified as RNA-like in 2004 and now found in RNA databases include several regulatory RNAs such as the GEMM cis-regulatory element (Rfam:RF01051, tree graph ID: (6,1)), the Tobamovirus internal ribosome entry site (Rfam:RF00225, tree graph ID: (8,2)), the mammalian CPEB3 gene (Rfam:RF00622, dual graph ID: (3,2)), the Tymovirus tRNA-like 3' UTR element (Rfam:RF00233, dual graph ID: (4,1)), and the Tombusvirus 3' UTR region IV (Rfam:RF00176, dual graph ID: (4,2)). RNAs such as the SAM riboswitch (Rfam:RF00162, tree graph ID: (6,5)), the Togavirus 5' cis-regulatory element (Rfam:RF00470, tree graph ID: (8,14)), and the viral 3' UTR (Pseudobase++: PKB169, dual graph ID: (4,29)) were misclassified as non-RNA-like topologies in 2004. Figures 4 and 5 show a more comprehensive selection of newly-confirmed topologies for tree and dual graphs, respectively, with corresponding second eigenvalues, secondary structures, topological depictions, and functionalities. Figure 5a shows the five identified RNAs that correspond to our candidate topologies with 3 and 4 vertices in 2004 using PAM [32] (C1, C2, C3, C4 and C7). Often, multiple sequences correspond to a graph (for example, 6, 4 and 2 sequences correspond to dual graphs (4, 1), (4, 2) and (4, 29), respectively). Twenty-two out of 71 existing dual graphs correspond to only one sequence. We classify all these cases as existing topologies.

### Assessment of *k*-NN versus PAM clustering methods

As discussed above, PAM clustering does not employ existing data to weigh the results: PAM selects two representatives and assigns each graph into the closest group among the two groups based on the Euclidian distance matrix of graph descriptors. These steps are repeated until the resulting two groups of graphs minimize the total distance between members in each group as well as maximize the distance between the groups. The members in the group containing more existing graphs are classified as RNA-like topologies.

We now employ instead a supervised *k*-nearest neighbor (*k*-NN) algorithm in conjunction with our RAG update. In the *k*-NN classification, missing motifs are assigned to RNA-like or non-RNA-like topologies based on *k *closest training data points of existing RNAs; the distances are measured by graph descriptors. The *k*-NN approach classifies an object according to a majority vote that depends on the object's neighbors: the object is assigned to the class most common among its *k *nearest neighbors. Thus, no medoids per se are defined as in PAM, and the classification changes as the data set of known RNAs increases. Our reclassification thus yields revised predictions of topologies that are more strongly guided by existing data.

To compare the performance of PAM and supervised clustering in terms of predicting RNA-like topologies, we use a standard statistical procedure, 10-fold cross-validation, in which the data are partitioned into 10 subsets, one of which is used as a training set and the others are used for prediction. Such a procedure is repeated by shuffling the data in various ways, and all predictions are averaged over these rounds. We perform 10-fold cross-validation for *k*-NN (with *k *= 1 to 5) and PAM clustering based on different graph sets using the R statistical package [39].

Table 3 shows that the error rate for classification of dual graphs from 3 to 9 vertices is 6-15% for *k*-NN compared to 26-36% for PAM. We find that *k *= 3 is a good choice for the former method (see Table 3). The trend is similar for tree graphs (Table S3 in Additional file 1).

In addition, we use partial sets (2004 data set plus 50% of 2010 data) to define other training sets and again compare the performance of *k*-NN. We have added more existing data to define these partial sets since the 2004 data set is too small on its own. Table 4 shows the results of dual graphs when the training set consists of 29 existing topologies in 2004, 21 additional existing topologies in 2010, and 50 missing graphs. Tested against all known existing motifs to date (21 new motifs), the accuracy is 95% (*k *= 1) to 81% (*k *= 5) compared to 62% for PAM. The trend is similar for tree graphs (72%-83% for *k*-NN and 77% for PAM, see Table S4 in Additional file 1).

**Table 4:** Prediction accuracy when partial sets consist of 2004 existing dual graphs and an additional 50% of 2010 data

Training Set	Testing Set	Method	Accuracy (%)
2004 Existing RNAs	50% of newly found	1-NN	95
		
Plus 50% of New RNAs	RNAs since 2004	2-NN	90
		
(29 existing in 2004, 21 new RNAs,	(21 new existing)	3-NN	90
		
and 50 missing dual graphs)		4-NN	90
		
		5-NN	81

No Training Set (all 71 existing and 53,739 missing graphs)		PAM	62

**Figure 6:**
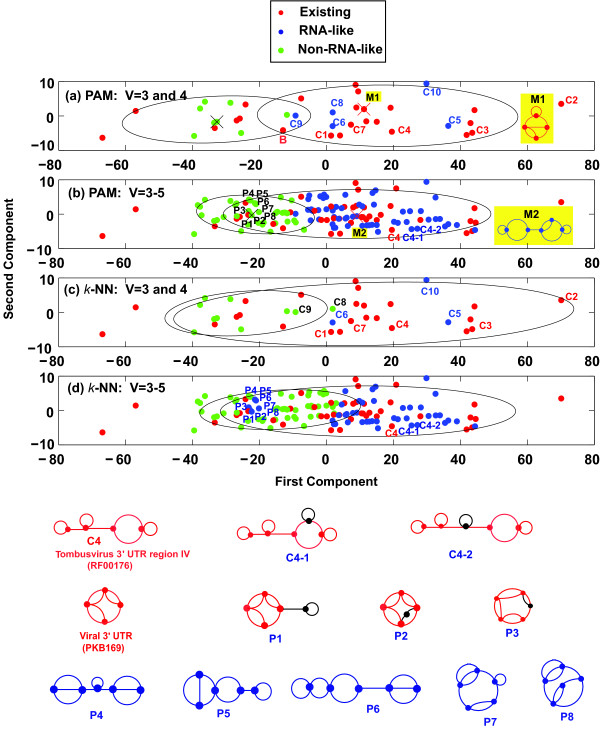
**Clustering plots of PAM and *k*-NN clustering for 38 RNA dual graphs with 3 and 4 vertices (a versus c) and for 146 RNA dual graphs with 3 to 5 vertices (b versus d)**. Existing, RNA-like, and non-RNA-like topologies are represented as red, blue, and green, respectively. Each ellipse encloses at least 85% of the RNA-like or non-RNA-like group members. In (a) and (b), the centers of two groups (M1 and M2) are marked with an X and the topologies of RNA-like groups' centers are shown. M1 and M2 emerge as pseudoknot RNAs with universal features, corresponding to structures of tmRNA (PKB234) and a candidate topology similar to the Box H/ACA snoRNA (RF00233). In (a), C1, C2, C3, C4, and C7 are existing topologies which were classified as RNA-like topologies in 2004 using PAM [32] (see Figure 5) and B is a confirmed bridge structure corresponding to U5 spliceosomal RNA (RF00020, see the second row in Figure 2). In (b) and (d), two candidate topologies (C4-1 and C4-2) are shown which are similar to the newly-confirmed existing topology C4 (Tombusvirus 3' UTR region IV). In (c), C5, C6 and C10 remain as RNA-like while C8 and C9 have been changed into non-RNA-like topologies. See Figures 5 and 9 for C1-C10. In (d), eight reclassified candidate pseudoknot topologies (P1-P8, currently RNA-like topologies which were classified non-RNA-like in 2004) are shown; P1, P2 and P3 are similar to the newly-confirmed existing topology Viral 3' UTR (Pseudobase++: PKB169).

Figure 6 compares the PAM and *k*-NN clustering of 146 dual graphs up to 5 vertices. PAM clusters 146 topologies without prior information of existing RNAs and predicts 61 to be RNA-like (0, 5 and 56 topologies with 3, 4 and 5 vertices, respectively), while *k*-NN reclassifies 48 topologies as RNA-like (0, 3 and 45 with 3, 4, and 5 vertices, respectively) (Table 2) based on existing RNAs with 3 and 4 vertices (25 existing topologies). Of the RNA-like topologies, 37 PAM classifications remain unchanged. Figure 6 also shows two examples of RNA-like graphs (C4-1 and C4-2, blue dots in both Figures 6b and 6d) that have similar structures to a confirmed candidate (C4, Tombusvirus 3' UTR region IV, RF00176, See Figures 5 and 9 for topologies). Eight of the 45 RNA-like topologies classified by *k*-NN were predicted to be non-RNA-like by PAM (P1-P8 in Figure 6d). Some of these eight topologies are similar to newly-confirmed topologies. Three new candidate pseudoknots (P1-P3) correspond to a substructure of Viral 3' UTR (Pseudobase++:PKB169) with an added stem. All of the newly classified RNA-like and non-RNA-like topologies are provided on our web resource http://www.biomath.nyu.edu/rna.

### Reclustering of RNA topologies and comparison of 2004 and current predictions

Our analyses above suggest that the supervised clustering algorithm can better take advantage of newly-confirmed topologies to lower the error rate and increase the accuracy in suggesting candidate RNA-like topologies. Our classification using the supervised *k*-NN algorithm applied to all existing RNA data is shown in Tables 1 and 2. When compared to predictions in 2004, the number of RNA-like graphs increases slightly for tree graphs (from 111 to 126, the last row in Table 1) and decreases more significantly for dual graphs (from 36,571 to 16,658, the last row in Table 2).

### The Updated RAG Database

**Figure 7:**
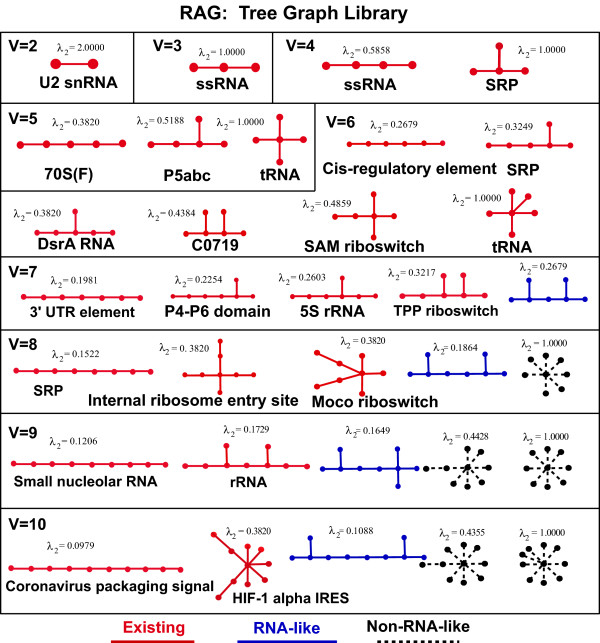
**The library of topologies for tree graphs between 2 and 10 vertices, with the second smallest Laplacian eigenvalue (λ_2_) listed**. RNA families with sequences belonging to select topologies are listed below their corresponding tree graph. Existing topologies, RNA-like, and non-RNA-like topologies based on the 2010 clustering are represented by red, blue or black (dashed) colors, respectively. For tree graphs with 2-6 vertices, the complete library is shown (all topologies are currently existing in nature). For tree graphs with 7-10 vertices, the partial library of topologies (existing, RNA-like, and non-RNA-like) is shown. See the RAG website for the complete library of topologies with 7-10 vertices.

The RAG update incorporates newly-confirmed topologies and new predictions. Figure 7 depicts updated information of tree topologies up to 6 vertices, as well as a partial sampling of existing, RNA-like, and non-RNA-like topologies for 7 vertices and up. RNAs corresponding to the full tree graph library up to 6 vertices (13 topologies) have been discovered in nature. Structures representing these newly confirmed topologies have functional roles as riboswitches, cis-regulatory elements, and tRNA, among many others. The full tree graph library can be viewed on our RAG website. Figure 7 also provides insights into the organization of RAG; each of the graphs within a certain vertex number is listed in order of increasing complexity, as determined by their second eigenvalues. As expected, multiple sequence and structure families map onto the same graphs. Also, there is a many-to-one relationship between sequence and motif. As an example, in Figure 7, for the existing topology with 6 vertices and a second eigenvalue (λ_2_) of 0.3249, we originally found, in addition to a signal recognition complex, more than 20 other regulatory non-coding RNAs corresponding including OxyS (Rfam:RF00035), SraG (Rfam:RF00082), GadY (Rfam: RF00122), t44 (Rfam: RF00127), and SL2 (Rfam: 00199). Note that because some of the structures available in 2004 were not readily classified in databases of the time, our updated search reports more overall topologies from the years leading up to 2004 than were reported in the original RAG paper.

**Figure 8:**
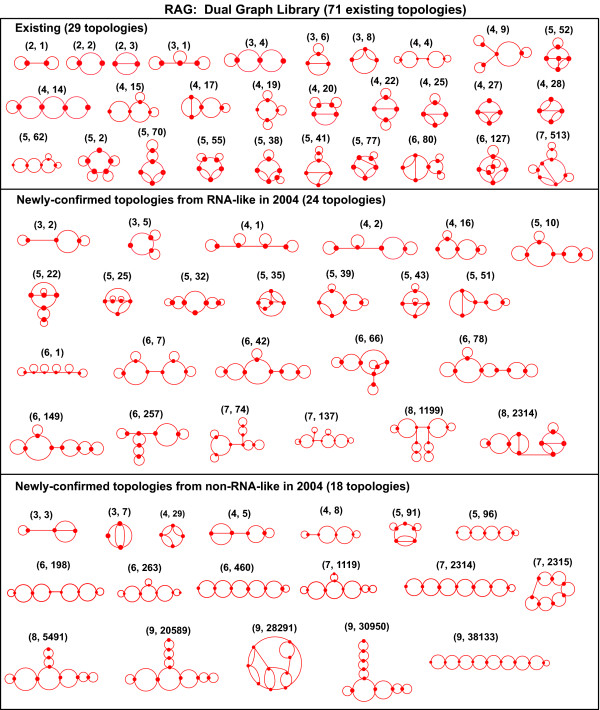
**The library of 71 existing topologies for dual graphs between 2 and 9 vertices: from these 71 topologies, 29, 24, and 18 were classified as existing, RNA-like and non-RNA-like, respectively, in 2004**. The vertex number and ID are in parentheses above each graph.

**Figure 9:**
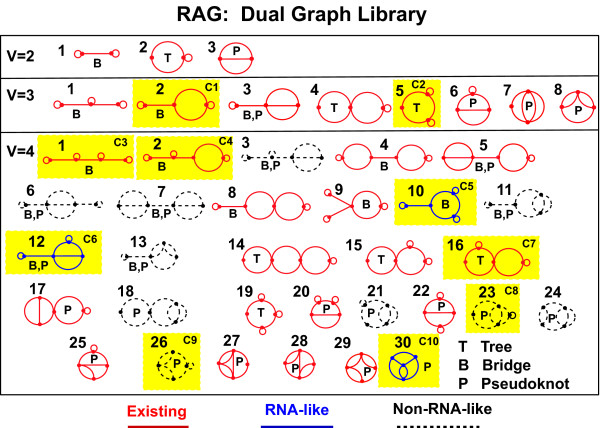
**The full library of topologies for dual graphs between 2 and 4 vertices**. The current status of existing, RNA-like, and non-RNA-like topologies is represented by red, blue or black (dashed) colors, respectively, based on the new clustering approach. The structural class (tree, bridge or pseudoknot) is also shown for each graph. Ten candidate topologies (C1-C10) in 2004 suggested by PAM are shown in the yellow box. See the RAG website for the library of topologies with 5-9 vertices.

All 11 dual graph topologies for up to 3 vertices have now been discovered. The complete repertoire of newly-confirmed topologies can be seen in Figure 8. Interestingly, several of these confirmed topologies consist of submotifs that were confirmed in 2004, linked by single stranded bridge regions. For example, the topology corresponding to the dual graph (6,78) is a combination of the (2,2) graph and the (4,15) graph via a single stranded region. This observation, along with our discovery of designed sequences from 2004, lends support to our modular design of candidate sequences. Figure 9, which contains the full dual graph library up to 4 vertices, shows some of these reclustered topologies, as indicated by blue (RNA-like) and black (non-RNA-like) coloring, along with their structural classifications (tree, bridge, or pseudoknot).

### Searching for functionality and its implications for RNA design

We have applied our RNA graph classification and prediction to RNA structure analysis and design in multiple ways. For example, we have applied our graph classifications to reveal modular RNA architectures by computational analysis of existing pseudoknots and ribosomal RNAs using dual graph isomorphism [40] and discovered motifs corresponding to antibiotics-binding aptamers in genomes by searching our graphs [41]. We have also assessed topological distributions of random pools for *in vitro *selection based on RAG [42]. Such combinations can be applied to *in vitro *selection in conjunction with our RAGPOOLS web server http://rubin2.biomath.nyu.edu/ to design new RNA pools with desired topologies [43-45].

Other groups have extended RAG to labeled dual graphs and directed tree graphs, including graph applications to non-coding RNA classification [18-22]. For example, the Brenner group has added labels to our dual graphs, enabling them to construct more detailed models of RNA structures that classify non-coding RNA families [18]. The Asai group has modified our tree graphs to include direction, which has allowed them to predict non-coding RNAs [19]. Heitsch and coworkers used tree graphical representation to analyze the branching degree of entire RNA viral genomes like Hepatitis C (9,400 bases) and, in turn, proposed a new pattern of random tree degrees in graph theory [46,47]. Knisley and coworkers extended our graph classification by developing more parameters for tree descriptors and providing a quantitative analysis of secondary structure of RNAs [48,49].

Our RAG update includes various features such as expanded search tools, directed/labeled graph graphs, a subgraph search program, and newly suggested RNA-like topologies. We hope that these improved features will allow users to perform complex queries and apply our resource to RNA design and related problems. In particular, users can use our updated RAG in four significant ways. First, researchers can translate RNA secondary structure into tree and dual graphs without length limitations on our web server http://www.biomath.nyu.edu/rna/analysis/rna_matrix.php. For example, a set of long viral RNAs (~1000 nt) can be translated into tree and dual graphs. Second, neighboring topologies are related to existing ones by size, topology or function and can be explored, which will help to search a family of topologies. Third, new candidate RNA-like topologies can be used for searching genomes. Fourth, RNA graphs can help design new classes of structural RNAs by combining multiple graphs.

### Software features

The updated version of RAG includes a program that converts a given secondary structure (in either 'ct' or 'bpseq' format) into a dual graph. Like the version of RNA Matrix for tree graphs released in 2004, users can submit a single secondary structure file for analysis. Using this file, we compute the necessary Laplacian eigenvalues and provide the corresponding dual graph topology, along with labeled vertices and directional information. The adjacency, degree, and Laplacian matrices for the structure are displayed and the common subgraphs between two structures can be evaluated. In addition, a set of secondary structures (up to 1000 ct files) can be submitted to the updated RNA Matrix program for batch processing and the resulting adjacency matrices and graph IDs can be downloaded.

We have changed our back-end database from plain text files into a MYSQL relational database implementation; the relational database tables in RAG have been "normalized" to minimize the redundancy and define relationships between them. By requiring the existence of a related row in another table, we improve the database integrity and make sure that the data entered into the database are valid and consistent. The updated database using a relational database thus allows us to provide different views and more advanced queries for users (e.g., sequence searches by Rfam ID). We plan to update RAG by adding new structures reported from publications or structural databases regularly, so that new information will become accessible on each topology's sub-page. We also invite users to submit their structures to us directly for inclusion in RAG.

We have also modified the front-end code to produce a better user interface; we have changed the format from static pages written in HTML and PHP server-side scripting languages into a rich-Internet compatible interface utilizing Web 2.0 features such as Asynchronous JavaScript and XML (AJAX) techniques.

## Conclusions

An improved and updated RAG database has been designed to allow experimentalists and theoreticians to explore currently existing motifs and help suggest novel RNA motifs. The number of available structures has increased, the searching capabilities have been improved, and the web server has a more user-friendly interface and dynamic content.

Because of the translation to a combined PHP and MYSQL interface, future updates of RAG will be accessible via the web resource in real time; each tree and dual graph topology has its own sub-page on the web resource that accesses the database and displays all structures corresponding to that particular topology. Thus, each addition to RAG will be displayed instantaneously on its respective online topology page.

The hypothetical 'RNA-like' topologies predicted by clustering techniques may serve as possible candidates for RNA design. Here we improved the clustering predictions made in 2004 [1,32] by using a *k*-NN clustering approach (Tables 1 and 2).

## Availability and Requirements

The RAG database, documentation, and software can be accessed on our web server http://www.biomath.nyu.edu/rna. We invite users to experiment and report to us their experiences.

## Authors' contributions

JAI and NK collected and analyzed data as well as wrote this paper. SE implemented the web interface and the relational database schema. TS designed and supervised the project including writing and editing the manuscript. All authors read and approved the final manuscript.

## Supplementary Material

Additional file 1 **Supplementary Tables S1-S4**. This additional file include the list of Rfam ID whose comparative structure is confirmed by mutagenesis or structure probing (Table S1), correction of the 2004 existing data (Table S2), cross-validation results for tree graphs (Table S3), and tree graphs classified by partial sets (Table S4). Click here for file
