# Supplementary material for: RAG: An update to the RNA-As-Graphs resource
Source: BMC Bioinformatics. 2011 May 31;12:219. doi: 10.1186/1471-2105-12-219 (PMC3123240; doi:10.1186/1471-2105-12-219)
Supplement: Additional file 1 — Supplementary Tables S1-S4. This additional file include the list of Rfam ID whose comparative structure is confirmed by mutagenesis or structure probing (Table S1), correction of the 2004 existing data (Table S2), cross-validation results for tree graphs (Table S3), and tree graphs classified by partial sets (Table S4). [file 1471-2105-12-219-S1.DOC]

**Supplementary Information**

J. A. Izzo *et al*., RAG: An Update to the RNA-As-Graphs Resource

**Table S1.** List of Rfam ID whose comparative structure is confirmed by mutagenesis or structure probing.

| RF00001 | RF00163 | RF00242 | RF00444 | RF00514 |
| --- | --- | --- | --- | --- |
| RF00002 | RF00164 | RF00250 | RF00453 | RF00515 |
| RF00003 | RF00165 | RF00259 | RF00458 | RF00524 |
| RF00008 | RF00167 | RF00260 | RF00460 | RF00548 |
| RF00012 | RF00168 | RF00264 | RF00462 | RF00550 |
| RF00013 | RF00169 | RF00286 | RF00463 | RF00552 |
| RF00014 | RF00171 | RF00290 | RF00467 | RF00615 |
| RF00015 | RF00172 | RF00348 | RF00478 | RF00616 |
| RF00019 | RF00175 | RF00362 | RF00480 | RF00617 |
| RF00020 | RF00176 | RF00363 | RF00481 | RF00618 |
| RF00021 | RF00179 | RF00364 | RF00483 | RF00619 |
| RF00022 | RF00180 | RF00365 | RF00484 | RF00622 |
| RF00025 | RF00181 | RF00366 | RF00485 | RF00626 |
| RF00026 | RF00182 | RF00367 | RF00487 | RF00632 |
| RF00031 | RF00183 | RF00378 | RF00489 | RF00634 |
| RF00032 | RF00184 | RF00381 | RF00490 | RF01046 |
| RF00037 | RF00192 | RF00382 | RF00491 | RF01047 |
| RF00050 | RF00194 | RF00383 | RF00492 | RF01051 |
| RF00059 | RF00196 | RF00384 | RF00493 | RF01053 |
| RF00065 | RF00198 | RF00385 | RF00494 | RF01054 |
| RF00094 | RF00199 | RF00386 | RF00496 | RF01055 |
| RF00102 | RF00207 | RF00388 | RF00498 | RF01057 |
| RF00106 | RF00214 | RF00389 | RF00499 | RF01065 |
| RF00107 | RF00215 | RF00390 | RF00500 | RF01066 |
| RF00109 | RF00220 | RF00391 | RF00502 | RF01067 |
| RF00114 | RF00231 | RF00433 | RF00505 | RF01068 |
| RF00128 | RF00233 | RF00434 | RF00506 | RF01069 |
| RF00140 | RF00234 | RF00435 | RF00507 | RF01070 |
| RF00161 | RF00236 | RF00436 | RF00512 | RF01116 |
| RF00162 | RF00238 | RF00437 | RF00513 | RF01410 |
| RF01411 |  | | | |

**Table S2.** Correction of the 2004 existing data. As a result of our current database search, the number of existing RNA topologies has been changed from 24 to 21 for tree graphs and from 30 to 29 for dual graphs.

| **Graph** | **(Vertex, ID)** | **Status as of 2004** | **Current status** | **Reason** |
| --- | --- | --- | --- | --- |
| Tree | (8, 15) | Existing  (5S rRNA database, predicted by Mfold) | Non-RNA-like | The structures of the 5S rRNA database could not be reproduced, but were produced using Mfold. |
| Tree | (9, 13) | Existing  (5S rRNA database, predicted by Mfold) | RNA-like |
| Tree | (10, 70) | Existing  (5S rRNA database, predicted by Mfold) | RNA-like |
| Dual | (5, 7) | Existing  (HDV ribozyme, PKB75) | RNA-like | The ct file for PKB75 corresponds to the dual graph (4, 25). |

**Table S3. Cross-validation (CV) results for tree graphs (***k***-NN).** We validate our *k*-NN (with *k*=1 to 5) clustering using cross-validation analysis. We use 10% (10-fold) of the data as the training set and the remaining 90% of the data as the testing set where the total consists of all existing RNAs with vertices from 3-7 to 3-10.

| Training Set (2010) | | Error Rate  (10-fold CV) [%] |
| --- | --- | --- |
| V | *k* |  |
| 3-7  (21 existing and 21 missing graphs) | 1 | 29 |
|  | 2 | 31 |
|  | 3 | 29 |
|  | 4 | 33 |
|  | 5 | 31 |
|  | PAM | 50 |
| 3-8  (36 existing and 36 missing graphs) | 1 | 36 |
|  | 2 | 29 |
|  | 3 | 36 |
|  | 4 | 31 |
|  | 5 | 33 |
|  | PAM | 39 |
| 3-9  (47 existing and 47 missing graphs) | 1 | 43 |
|  | 2 | 44 |
|  | 3 | 39 |
|  | 4 | 38 |
|  | 5 | 35 |
|  | PAM | 50 |
| 3-10  (57 existing and 57 missing graphs) | 1 | 44 |
|  | 2 | 48 |
|  | 3 | 39 |
|  | 4 | 48 |
|  | 5 | 43 |
|  | PAM | 40 |

**Table S4. Tree graphs classified by partial sets (2004 data plus 50% of 2010 data).**

| Training Set | Testing Set | Method | Accuracy (%) |
| --- | --- | --- | --- |
| 2004 Existing RNAs  Plus 50% of New RNAs  (21 existing in 2004, 16 missing graphs and 19 new RNAs) | 50% of newly found RNAs since 2004  (18 new existing) | 1-NN | 72 |
|  |  | 2-NN | 83 |
|  |  | 3-NN | 83 |
|  |  | 4-NN | 89 |
|  |  | 5-NN | 83 |
| No Training Set (All 58 existing and 142 missing graphs) | | PAM | 77 |
